# How Particle Deformability Influences the Surfactant Distribution in Colloidal Polymer Films

**DOI:** 10.1021/acs.langmuir.2c02170

**Published:** 2022-10-04

**Authors:** Toby R. Palmer, Hanne M. van der Kooij, Rohani Abu Bakar, Mathis Duewel, Katja Greiner, Callum D. McAleese, Pierre Couture, Matthew K. Sharpe, Richard W. Smith, Joseph L. Keddie

**Affiliations:** † Department of Physics, University of Surrey, Guildford, SurreyGU2 7XH, United Kingdom; ‡ Physical Chemistry and Soft Matter, Wageningen University & Research, 6708 WEWageningen, The Netherlands; § Synthomer Germany GmbH, Werrastraße 10, 45768Marl, Germany; ∥ Surrey Ion Beam Centre, University of Surrey, Guildford, SurreyGU2 7XH, United Kingdom

## Abstract

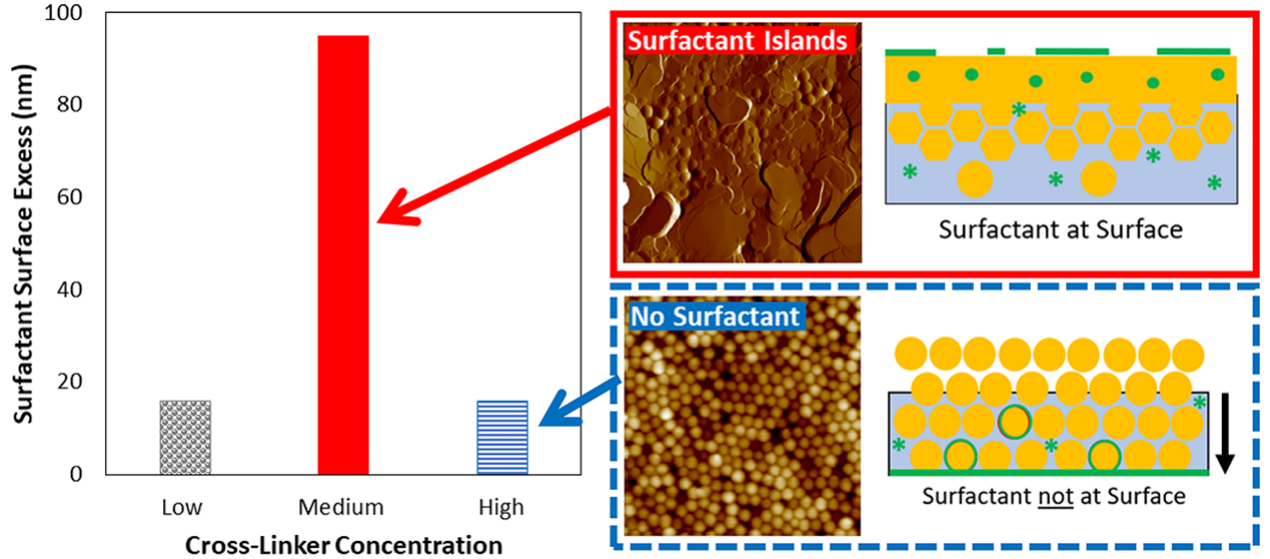

The distribution of surfactants in waterborne colloidal polymer films is of significant interest for scientific understanding and defining surface properties in applications including pressure-sensitive adhesives and coatings. Because of negative effects on appearance, wetting, and adhesion, it is desirable to prevent surfactant accumulation at film surfaces. The effect of particle deformation on surfactant migration during film formation was previously investigated by Gromer et al. through simulations, but experimental investigations are lacking. Here, we study deuterium-labeled sodium dodecyl sulfate surfactant in a poly(butyl acrylate) latex model system. The particle deformability was varied via cross-linking of the intraparticle polymer chains by differing extents. The cross-linker concentration varied from 0 to 35 mol % in the copolymer, leading to a transition from viscoelastic to elastic. Ion beam analysis was used to probe the dry films and provide information on the near-surface depth distribution of surfactant. Films of nondeformable particles, containing the highest concentration of cross-linker, show no surfactant accumulation at the top surface. Films from particles partially deformed by capillary action show a distinct surfactant surface layer (ca. 150 nm thick). Films of coalesced particles, containing little or no cross-linker, show a very small amount of surfactant on the surface (ca. 20 nm thick). The observed results are explained by considering the effect of cross-linking on rubber elasticity and applying the viscous particle deformation model by Gromer et al. to elastically deformed particles. We find that partially deformed particles allow surfactant transport to the surface during film formation, whereas there is far less transport when skin formation acts as a barrier. With elastic particles, the surfactant is carried in the water phase as it falls beneath the surface of packed particles. The ability to exert control over surfactant distribution in waterborne colloidal films will aid in the design of new high-performance adhesives and coatings.

## Introduction

Colloidal polymer films have numerous applications, including pressure-sensitive adhesives (PSAs) and coatings. They can be deposited from polymer colloids dispersed in water (latex), which reduces the emission of environmentally damaging organic solvents when compared with traditional solvent-cast films.^1^ The manufacturing of latex for PSAs and coatings via emulsion polymerization requires the use of surfactants, which aid in the synthesis and impart colloidal stability.^2,3^ PSAs adhere to nearly any surface under the application of light pressure and have applications ranging from tapes, labels, and bandages to graphics and mechanical joints in aircraft.^4^ Unfortunately, surfactants have been seen to cause detrimental effects, in particular on the properties of PSAs,^5^ such as a reduction in their adhesion strength,^6−9^ blocking of particle coalescence,^10^ and a loss of optical clarity caused by water whitening.^11^ These problems typically occur most prominently when surfactant accumulates at the surfaces of films. Being able to control the distribution of surfactants near the surfaces of such films is essential for the design of high-performance adhesives (and coatings) that will not experience previously seen performance losses.

The nonuniform distributions of surfactants, and their tendency to accumulate at film interfaces, have been extensively studied,^12,13^ with research showing it gathers in greater amounts at the substrate interface^14^ in some cases, and at the air interface in other cases.^15^ Broadly, two classes of operative mechanisms have been described. Surfactants in colloidal films can accumulate at interfaces *during* the water evaporation process, *or* they can be *exuded* over time after the completion of film formation. The former is the mechanism studied in this work.

Mallegol et al.^10^ showed that surfactant can prevent complete particle coalescence when it accumulates between the particles near the surface, but upon rinsing with water, the excess surfactant removal allows for rapid particle coalescence. The enrichment has also been seen to increase over time, with the kinetics of surfactant exudation being reported to depend on the type and size of surfactant molecules,^16^ as well as the temperature and humidity of the environment.^17^ The type of surfactant and its concentration have similarly been shown to influence the tendency for accumulation at the film–substrate interface during film formation.^18^

### Models of Latex Film Formation and Surfactant Distribution

The film formation of latex colloids has been well studied and quantified. In the deformation step of film formation, spherical latex particles are deformed to fill the space between neighboring particles. A general consensus has now been reached outlining four possible mechanisms for the deformation process. The pioneering work of Routh and Russel^19,20^ set out a clear deformation map that can be used to identify the deformation regime of a particular system, as a function of several key parameters. Two dimensionless numbers are used in the map: a deformation ratio, λ, and the Péclet number, *Pe*. Both numbers are shown to be dependent on the evaporation rate of water, *E*, and the initial wet film thickness, *H*. λ is used to describe the competition between the deformation rate of *viscous* polymer particles (with a radius of *R* and zero-shear-rate viscosity of η_0_) and the evaporation rate, *E*. The parameter is defined as
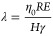
1where γ represents the interfacial tension of the relevant interface driving particle deformation (polymer/air; polymer/water; or water/air). Routh and Russel proposed using a typical value of 0.07 N/m to capture the correct order of magnitude for λ.^20^

In the regime 0 < λ < 1, the time for particle deformation to occur is less than the time for evaporation to reduce the water level of the surface. The wet particles are deformed when still in water, to reduce the polymer/water interfacial energy. This is called the wet sintering regime, as was first proposed by Vanderhoff et al.,^21^ with experimental evidence available from several authors.^22−25^ For 1 < λ < 100, capillary deformation acts as the driving force.^26−28^ Compressive capillary pressure caused by the water meniscus between the particles at the surface is greater than the stress required to deform the particle network. The water evaporation and particle deformation occur simultaneously. In the case of elastic spheres with a shear modulus, *G*, when viscous flow does not occur,^29^ several teams of researchers^21,26,30^ derived the condition for particles to fill space entirely under capillary action. According to the original work by Brown et al., this condition must hold:
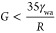
2where γ_wa_ refers to the water/air interface. The next regime is the receding water front, in which 100 < λ < 10 000.^23,31^ This is an inhomogeneous regime in which the initial particle deformation occurs due to capillary forces; however, the final coalescence of the particles occurs after the water front has dropped below the particles, under the dry sintering regime. Finally, for λ > 10 000, particles are deformed in the absence of water, driven by the reduction in the polymer/air interfacial energy.^32,33^ The time for particles to deform is longer than for water to evaporate. This is called the dry sintering regime.^20^

To model the distribution of the various particles in the vertical direction of a drying colloidal film, a Péclet number, *Pe*, has been defined. *Pe* describes the competition between the descending top surface of a drying film that sweeps up particles as water evaporates and the self-diffusion of the particles that will redistribute them in the continuous water phase with a viscosity of μ. The diffusion coefficient of the particles is generally given by the Stokes–Einstein equation:
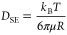
3where *k*_B_ is the Boltzmann constant, and *T* is the absolute temperature. *Pe* for the polymer particles is given by
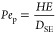
4For the case that *Pe*_p_ ≫ 1, diffusion is slow compared to the evaporation of water, and particles will become trapped at the film/air interface during drying. When in the regime where the particles are soft (low λ), there will be the formation of a coalesced skin layer on the top surface of the drying film.^34−37^ For the case that *Pe*_p_ ≪ 1, diffusion is fast compared to the evaporation water, and so particles will be able to reduce concentration gradients, giving a more homogeneous film structure during drying. A similar *Pe* can be used to describe the water-soluble surfactants, *Pe*_s_, using the self-diffusion coefficient of surfactants in solution.

Previous experimental work tested the validity of the Routh-Russel deformation model.^36,38^ Gonzalez et al.^36^ systematically changed η_0_, *E*, and *H* and examined the effect on the structure of the film during formation. They found evidence for different deformation mechanisms that could be explained through trends in estimated values of λ. Carter et al.^38^ used GARField NMR profiling to show the water distribution in films for different values of λ (obtained by varying the polymer’s glass transition temperature, *T*_g_) and *Pe* and identified film formation by wet sintering (and skin formation), capillary deformation, and dry sintering mechanisms. Although this research analyzed the distribution of water during film formation, it did not consider effects on the surfactant.

Inspiring our present experimental research, Gromer et al.^39^ proposed a computational model that simulates the behavior of polymer particles in a drying film, as a function of λ and *Pe*, followed by their work studying the vertical surfactant distribution in a drying latex film.^40^ They used the model by Routh and Russel as a bedrock and built upon the computational modeling of surfactant distributions in latex films by Gundabala et al.^41^ The Gromer model uses cellular automata and divides the vertical orientation into discrete cells that each contain a unique particle composition. The model describes the transfer of particles and surfactant between these cells. With their model, they simulated the surfactant distribution during film formation in three of the deformation regimes: wet sintering, capillary deformation, and receding water front.

**Figure 1:**
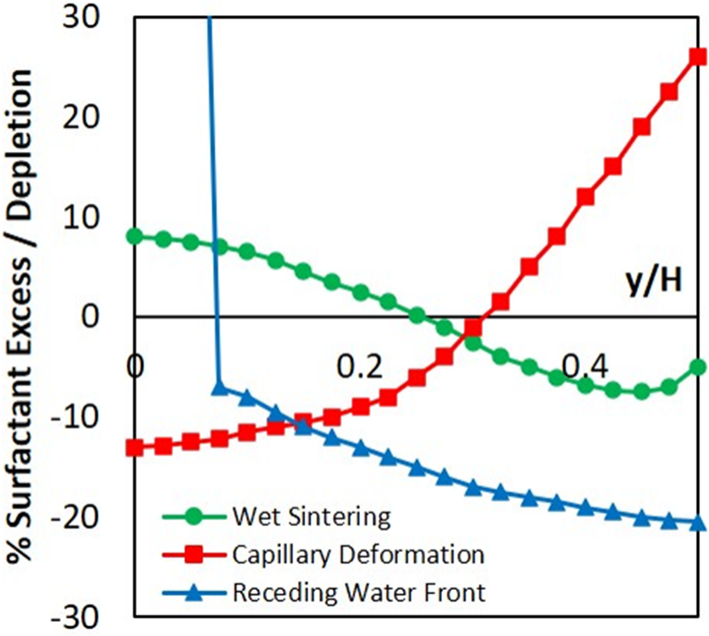
Surfactant distribution as a function of the vertical height, *y* (measured from the bottom substrate interface, for which *y*/*H* = 0), normalized by the initial thickness of the wet film, *H*, for different particle deformation mechanisms: wet sintering (λ = 0.5); capillary deformation (λ = 25); and receding water front (λ = 200). All profiles are shown for a normalized drying time of 1.49, at which time the particle volume fractions had reached ∼0.9. A normalized drying time of 1 corresponds to the time when the particle volume fraction has reached ϕ = 0.64 (random close-packing) in contact with the substrate. For the particles, *Pe*_p_ = 50; for the surfactant, *Pe*_s_ = 0.5. Adapted with permission from ref (40). Copyright 2017 American Chemical Society.

Figure 1 shows a summary of their results. In the wet sintering regime (λ = 0.5), it was observed that when the particles had been close packed, there was surfactant depletion near the top air/film interface, and an excess concentration near the substrate interface. The profile is explained by considering skin formation causing surfactant desorption from the particles accumulating at the top surface, thus leading to a depletion of surfactant at the top of the film. The concentration gradient of desorbed surfactant in the continuous phase drives the downward diffusion of the surfactant toward the substrate. The upward transport of surfactant is inhibited by the reduced void space available between the soft deforming particles.

For capillary deformation (λ = 25), it was predicted that there will be a strong surfactant excess at the top surface following drying. In this scenario of the Gromer model, there is a much more uniform deformation of particles, leading to a uniform desorption of surfactant into the water phase as drying proceeds. The downward movement of the particle front displaces the water (and surfactant) to the top surface, and capillary flow of the water phase leads to a stronger surfactant excess at the top surface. Finally, in the receding waterfront regime (λ = 200), it was found that a strong surfactant excess is seen near the substrate interface of the film, with a depletion near the top surface. In this scenario, the particles are likely to remain spherical during drying, and any surfactant in the continuous water phase will be carried down to the bottom of the film, as the water front recedes during drying.

The model of Gromer et al.^40^ (which built on Gundabala et al.^41^) provides a good framework for systematically testing the effect of varying particle deformability on the surfactant distribution. The work presented here has used the cross-linking of polymer chains to change the particle deformation. Although the Gromer model was derived for particles undergoing viscous deformation, the concepts of the space-filling of particles apply equally well to elastic particles with differing elastic moduli.

Previous work has explored the effect of polymer cross-linking and *T*_g_ on the distribution of surfactant in films during both film formation and later stage exudation.^17,42,43^ It was found by Kessel et al.^17^ that films with *softer* (lower *T*_g_) particles develop a surfactant excess on the air surface approximately 5 nm thick, whereas those containing cross-linker saw no surfactant on the air surface, suggesting that the addition of cross-linker inhibits surfactant accumulation during film formation. Likewise, the work of Belaroui et al.^42^ showed that more surfactant accumulated at the interfaces for films with a lower *T*_g_, which is to say that films with softer particles (and therefore a lower λ, although this parameter was not considered) exhibit surfactant accumulation, while films with harder particles do not. Guigner et al.^43^ showed the tendency of surfactant to accumulate at the film/substrate interface during the film formation from cross-linked colloidal particles.

As this review has highlighted, processing conditions (especially the evaporation rate) and the type of surfactant (charge and diffusivity) will influence the distribution of surfactant in dry films. However, there is a gap in studying the sole effect of a changing λ on the distribution of surfactants in colloidal polymer films, in a clear, systematic way that isolates the change in λ, without changing other factors. Based on the work of Gromer et al.,^40^ we hypothesized that the space between the colloidal particles and its geometry will likely have an influence on the surfactant transport and distribution. In this work, we aimed to control the surfactant distribution through changes to the degree of polymer cross-linking in latex particles, which has the effect of changing their viscoelasticity.

Here, we have used ion beam analysis (IBA), including elastic recoil detection (ERD) and Rutherford backscattering spectrometry (RBS) to study the distribution of surfactant in colloidal polymer films as a function of the degree of cross-linking of the polymer chains. Previous work has shown the suitability of ERD^44^ and RBS^10,15,45^ for studying the composition as a function of depth of colloidal polymer films. We have used gravimetry to study the influence on the rate of water loss and employed mechanical analysis to measure the effect of cross-linking to provide estimates of λ. We have compared the surfactant depth profiles to the expectations of the model produced by Gromer et al.^40^

## Experimental Section

### Latex Synthesis and Sample Preparation

Poly(butyl acrylate) colloidal dispersions were synthesized using one-step emulsion polymerization. Full details are available in the publication by van der Kooij et al.^46^ Butyl acrylate (BA) served as the monomer, deuterated sodium dodecyl sulfate (d-SDS) as the surfactant, potassium persulfate (KPS) as the initiator, and ethylene glycol dimethacrylate (EGDMA) as the cross-linking agent. The colloidal dispersions typically had a solids content of ∼10 wt %. A series of latex was synthesized in which the EGDMA cross-linker concentration was varied systematically as 0, 1, 2, 5, 10, 15, 20, 25, and 35 mol %. The cross-linker concentration is calculated as *n*_EGDMA_/(*n*_EGDMA_ + *n*_BA_), where *n* is the number of moles. It was confirmed by van der Kooij et al.^46^ that, for films with 5 mol % cross-linker or more, the amount of non-cross-linked polymer is negligible, suggesting a gel content of 100%. This series presents a transition in application from viscoelastic particles at the low end of cross-linking (suitable for pressure-sensitive adhesives) toward elastic particles with the higher levels of cross-linking (making brittle coatings). Particle sizes were determined via dynamic light scattering (DLS). Measurements of the diluted samples were performed on a Malvern Zetasizer Nanoseries instrument (Nano S, ZEN1600), using a 4 mW 632.8 nm He–Ne red laser and avalanche photodiode detector measuring light intensity at a detection angle of 173°. The *z*-average values of the particle radius, *R*, are presented alongside values of the cross-linker concentration of the samples in Table 1.

**Table 1:** Particle Properties for the Series of Latex Particles

EGDMA cross-linker concentration (mol %)	density of EGDMA groups, ρ (10^19^ EGDMA/cm^3^)	particle radius, *R* (nm)	Pe_p_ in IBA experiment	glass transition temp, *T*_g_ (°C)
0	0.0	70	14	–46.4
1	3.6	67	14	–44.0
2	7.2	78	16	–42.3
5	22	96	20	–35.8
10	41	103	21	–21.5
15	60	96	19	0.7
20	79	145	29	14.4
25	99	117	24	18.1
35	130	152	31	–a

a A glass transition could not be detected.

Glass transition temperatures were determined using differential scanning calorimetry (DSC) using a TA Instruments Discovery DSC 250 instrument (Newcastle, DE). 75 μL of the wet samples (10 wt % solids) was drop cast into pans and dried on a hot plate at 60 °C, such that the dry polymer (mass of 6–8 mg) was analyzed. A heat/cool/heat cycle was used, with a heating rate of 20 °C min^–1^ over a range from −80 to 80 °C. The *T*_g_ was determined from the second heating curve.

There is a general upward trend in particle radius with increasing EGDMA content. We attribute any deviation from the trend to uncontrolled temperature fluctuations, especially during the particle nucleation stage.

The molecular weight of the 0 mol % EGDMA cross-linker particles was determined by gel permeation chromatography (GPC). GPC analysis was performed on a Viscotek GPCMax VE 2001 instrument, which has three linear columns (7.5 × 300 mm PLgel mixed-D) operating at 35 °C and a flow rate of 1.0 mL/min with tetrahydrofuran (THF) as a mobile phase. PMMA standards were used to calibrate the GPC. Before injection, samples (2–4 mg/mL) were dissolved in THF overnight and filtered through 0.2 μm regenerated cellulose syringe filters.

### Gravimetric Analysis of Evaporation

200 μm wet films were cast using a cube applicator for even coating onto acetone-cleaned glass plates (7.6 cm × 5.2 cm). Immediately following film casting, the samples were placed onto a digital balance (ENTRIS224I-1S, Sartorius, Goettingen, Germany) inside a humidity-controlled chamber. The humidity was held constant at 15% RH, using silica gel, and the temperature was 20.5 °C, as set by a temperature-controlled room. The balance was connected to a PC which provided a full readout of the mass of the film as a function of time, using WinWedge data collection software. Measurements continued for up to 4 h to ensure complete drying. A low humidity environment was chosen for practicality purposes since the dispersions have a solids content of approximately 10 wt % and as such take a long time to dry. For polymer particles, *Pe* was calculated using a continuous phase viscosity of 1 × 10^–3^ Pa s for the aqueous phase. For the SDS surfactant unimers and KPS initiator species, literature values are taken for their diffusion coefficients of 6 × 10^–10^ and 1 × 10^–9^ m^2^/s, respectively.^47,48^ This gives values for *Pe* as *Pe*_s_ = 0.01 and *Pe*_KPS_ = 0.004, with *Pe*_p_ presented in Table 1 showing the size dependence.

### Ion Beam Analysis (ERD and RBS)

For the purposes of ion beam analysis and atomic force microscopy measurements, additional surfactant (d-SDS) which was 5 wt % of the polymer mass was postadded to the as-synthesized samples prior to film casting. d-SDS (99% purity; Sigma-Aldrich) was first dissolved in deionized (DI) water and then stirred into the dispersions with a magnetic stirrer. The addition of surfactant was to aid in the analysis in both IBA and AFM, as a greater concentration of surfactant enables easier detection in both techniques due to greater signals.

With the additional level of surfactant present, the concentration of SDS in the system is 0.182 mol/L, which far exceeds the critical micelle concentration (CMC) for SDS, 0.0082 mol/L,^49^ and so surfactant in the dispersion will be in the form of micelles, rather than unimers. Micellization will have the effect of enlarging the size and slowing down the diffusion of the surfactant.

300 μL of the colloid/surfactant blends was drop cast using a micropipette on 2 cm × 2 cm silicon wafers and spread evenly to give an initial wet film thickness of *H* = 750 μm. Films were dried at 20.5 °C on a benchtop in a temperature-controlled room for a minimum of 24 h. These film formation conditions provided a value of *E* = 58 nm/s, as determined by gravimetry. A value for *D*_SE_ for the polymer particles was as it was for the gravimetry, and the diffusion coefficient for the initiator was also the same. For the surfactant, since we have micelles with a known radius taken from the literature of 1.5 nm,^50^ we can use the Stokes–Einstein equation to estimate *D*_SE_ as 1.4 × 10^–10^ m^2^/s, which is in broad agreement with experimentally measured values for the diffusion coefficient of SDS micelles.^51−54^ Estimates of *Pe* for the surfactant and initiator species are *Pe*_s_ = 0.3 and *Pe*_KPS_ = 0.04, with *Pe*_p_ presented in Table 1, for the films used in the ion beam analysis and subsequent atomic force microscopy.

Films were analyzed at the Surrey Ion Beam Centre by performing ERD and RBS simultaneously, with a 2 MeV ^4^He^+^ beam incident on the surface at an angle of 75° to the sample normal. The geometry is shown in Figure S1. The beam had a diameter of approximately 1 mm. A 6.5 μm thick aluminum range foil was used to filter out any forward scattered ^4^He^+^ ions that would otherwise be incident on the ERD detector. Spectra were acquired for a total charge of 10 μC for each sample. ERD and RBS spectra have both been analyzed and modeled using SIMNRA software,^55^ in which we use a simple (multi)slab model to fit the data to a given film structure. Pearson’s χ^2^ test was used as a measure of the goodness of fit of the model. Beam calibration parameters are provided in the Supporting Information. The data analysis considered the effect of H and D loss during the measurements, following the measurements in Figure S2.

### Atomic Force Microscopy

For AFM, the same samples as used during IBA were analyzed; therefore, the same sample preparation procedure applies. Images were recorded on a Bruker Dimension Edge with Scan Asyst atomic force microscope, using Bruker’s Scan Asyst image optimization technique. This technique is a type of peak force tapping, that requires minimal user input for parameters, such as the set point, since they are automatically adjusted by a feedback loop to optimize the image, based on the information received about the sample surface. For some samples, intermittent contact (tapping) mode was also used.

For Scan Asyst imaging, a SCANASYST-AIR silicon tip on a silicon nitride cantilever was used, with a nominal resonant frequency of 70 kHz and a nominal spring constant of 0.4 N/m, given by the manufacturer. For tapping mode imaging, an OTESPA-R3 silicon tip on a silicon nitride cantilever was used, with a nominal resonant frequency 300 kHz and a spring constant of 26 N/m, given by the manufacturer. Images were typically obtained using a scanning rate between 0.5 and 1 Hz.

### Additional Characterization

Methods for tensiometry of the latex and mechanical property characterization are provided in the Supporting Information.

## Results and Discussion

For this discussion, the samples have been broadly divided into low (<10 mol %), medium (10–15 mol %), and high (>15 mol %) levels of EGDMA cross-linker. The Gromer et al.^40^ model was developed for *viscoelastic* particles, but we propose that the surfactant transport in the space around viscously deformed particles will be comparable to that through equivalent geometric structures obtained via elastic particle deformation. To deduce the most likely particle deformation mechanisms, the elastic properties, film microstructure, and evidence for skin formation during drying were investigated.

### Polymer Viscoelasticity and Elasticity

The weight-average molecular weight for the pBA without cross-linker (0 mol %) was found to be *M*_w_ = 398 000 g/mol (with *Đ* = 1.93), which is far above the entanglement molecular weight of *M*_e_ = 25 000 g/mol,^56^ and its *T*_g_ = −46.4 °C. Thus, this entangled polymer is viscoelastic at room temperature. The gel fraction of the 1 mol % EGDMA particles was measured to be 80 wt % (via Sohlet extraction), and the *T*_g_ is only −44.0 °C (Table 1), which means that the particles with this level of cross-linking and higher can be classified as elastomeric at room temperature with some viscous dissipation from non-cross-linked chains. With additional cross-linking, a stiffening of the polymer is expected. In addition to the effect of restricting chain motion by the presence of cross-linking points, the copolymerization with EGDMA will itself harden the copolymer. There is a strong increase observed with increased cross-linking by EGDMA (Table 1), which is expected because poly(ethyl glycol dimethacrylate) has a reported *T*_g_ of 4 °C.^57^ At the maximum amount of EGDMA copolymerization (35 mol %), a glass transition could not be detected by DSC analysis. However, following the trend in *T*_g_ observed in Table 1, the 35 mol % EGDMA copolymer is assumed to have a cross-linked glass network at the temperature of film formation (20.5 °C). With the lower EGDMA concentrations, the network is expected to be elastomeric at room temperature.

**Figure 2:**
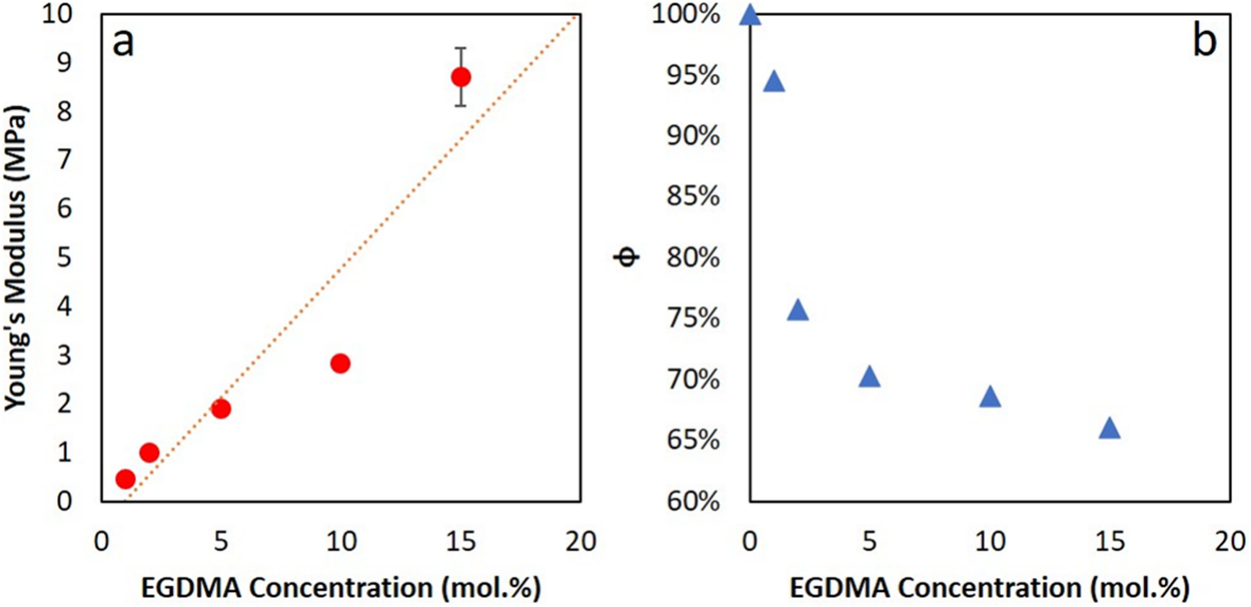
(a) Experimental values of the Young’s modulus as the cross-linker concentration is increased. Error bars represent the standard deviation associated with replicate measurements but are only visible for one sample. (b) Theoretical estimates of the space filled by the packing of elastic spheres using the measured Young’s modulus values for each cross-linker concentration.

The Young’s modulus, *Y*, was experimentally determined for samples with 15 mol % EGDMA cross-linker or less. Above this amount, free-standing films are too brittle to perform tensile testing, since the extensive cross-linking prevents interdiffusion of the polymer chains across particle boundaries, and there is no cohesion.^58^ For the 15 mol % EGDMA film, *Y* was measured using tensile testing. For films with 0–10 mol % EGDMA, the stress/strain relation from the probe tack analysis of films was used to find an estimate of the shear modulus, *G*, of the polymer in confinement. Using the relationship in eq 5, *Y* can be found. All experimental values are presented in Figure 2a, with the original data presented in Figure S3. As expected, the modulus increases linearly with increasing concentrations of EGDMA cross-linker.

We recall the criterion in eq 2 for the complete particle deformation of elastic particles by capillary action. Using an order of magnitude estimate of *R* = 100 nm and an experimental value for γ_wa_ obtained from pendant drop tensiometry of γ_wa_ = 25 mN m^–1^, we can obtain an upper limit for *G* that will allow complete space filling of particles under capillary pressure.

By assuming an isotropic solid such that
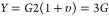
5with a value of 0.5 for the Poisson’s ratio, υ, of a noncompressible elastomer,^59^ we find that *Y* < 26 MPa will lead to complete capillary deformation. Our estimate indicates that capillary deformation will be operative for particles with an EGDMA concentration of approximately 15 mol % or lower in our series.

The particles’ elasticity will determine the extent of deformation during film formation. The volume of space filled, ϕ, is related to the strain deformation between spherical particle pairs in a packed bed, as presented by Routh and Russel.^19^ Using the equation for the strain from Johnson, Kendall, and Roberts^60^ for spheres with a shear modulus of *G*, ϕ can be written as
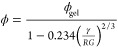
6where ϕ_gel_ is the packing fraction of hard spheres (taken here as random packing with a value of 0.65^61^). For the case of wet sintering, we take γ for the polymer/water interface to be 0.03 J m^−2^ as was used elsewhere for a similar system.^62^ This model neglects any viscous deformation of the spheres.

According to this simple elastic model, the volume fraction filled by the particles tends toward the value for that of random packing, ϕ = 0.65, as EGDMA concentration is increased (Figure 2b). For higher EGDMA concentration, particles are not predicted to deform significantly but remain spherical in shape. For lower EGDMA concentrations, there will be a certain amount of deformation, potentially producing narrow capillaries between particles and influencing water transport in the film. In the case of particles with low (1 mol %) or no (0 mol %) EGDMA cross-linking, there will additionally be substantial viscous flow of the particles, which will increase ϕ. Nevertheless, the key concept is that particles with low cross-linker concentrations will experience extensive deformation and ultimately coalesce to produce a cohesive film.

### Characteristic Drying Times and Skin Formation

For colloids in the wet sintering regime (with low viscosity), film-formed with *Pe*_p_ > 1, skin formation is expected. A signature of skin formation during the drying step is a slowing down of the rate of water loss. To investigate the effect of cross-linking on the rate of water loss from drying films, gravimetry experiments were performed, alongside AFM to study the surfaces of dry films.

**Figure 3:**
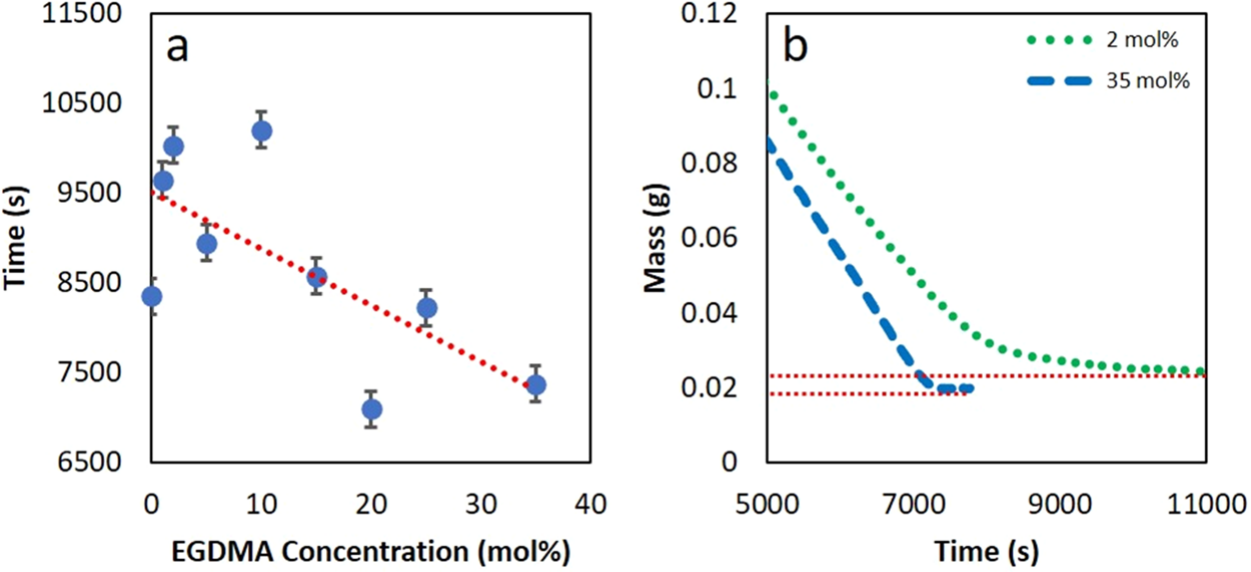
(a) Drying times as a function of EGDMA cross-linker concentration, as obtained from gravimetric analysis. (b) Raw data of mass versus time for the sample with low EGDMA cross-linker (2 mol %) and with the highest level of cross-linker (35 mol %). Error bars represent the uncertainty associated with defining the beginning and end of drying.

The characteristic drying time as a function of EGDMA concentration, obtained via gravimetric analysis, is presented in Figure 3a. These times are extracted from mass versus time curves, two examples of which (for 2 and 35 mol % EGDMA) are shown in Figure 3b. The point at which the differential of mass versus time curves reaches a value of 0 was used to estimate the characteristic drying time. The 35 mol % EGDMA sample shows a constant rate of water loss, with a nearly linear loss of mass over time until there is no water remaining. In contrast, the 2 mol % EGDMA sample starts off with a linear loss of mass, but it starts to slow at later times, indicating the formation of a skin layer of coalesced particles on the surface, which acts as a barrier to water loss from the film’s top surface.

Figure 3a shows that films with higher levels of cross-linker concentration tend to have a lower drying time. This result is most likely due to the extent of “openness” of the film structure, because particles with a higher elastic modulus will be deformed to a lesser extent to leave greater space for water transport (both liquid and vapor). Viscoelastic particles (with little or no cross-linker) are expected to be more extensively deformed to create a skin barrier for transport. The longer drying times at lower EGDMA concentrations are consistent with the formation of a skin layer with reduced permeability. According to the deformation map of Routh and Russel,^20^ a skin layer will be formed during film formation of particles that are within the wet sintering regime, for which λ < 1, and also when *Pe*_p_ > 1, leading to particle accumulation at the film surface.

To interpret the results quantitatively, we estimate a value of λ for the poly(butyl acrylate) particles (0 mol % EGDMA). Tobing and Klein^63^ used rheometry to measure the viscoelasticity of poly(butyl acrylate) with *M*_w_ = 263 000 g/mol at 25 °C as a function of the shear frequency. The *M*_w_ of the 0 mol % sample has the same order of magnitude. We take their viscosity value at a low frequency of 0.016 Hz to estimate the zero shear-rate viscosity, η_0_, to be 7 × 10^5^ Pa s. We use a typical value for γ_pw_ of 0.03 J m^−2^;^62^ the value for the particle radius, obtained from DLS, of *R* = 70 nm; and the measured evaporation rate of *E* = 7.6 × 10^–8^ m s^–1^. These values combine to give λ = 6 × 10^–4^ for the gravimetric experiment, which is far below 1 and so certainly within the wet sintering regime, as was defined by Routh and Russel.^20^

The gravimetry data also provide values of evaporation rate, *E*, to allow estimates of *Pe*_p_ and *Pe*_s_ to be made for other experiments under the same conditions. The range of *Pe*_p_ is 5 < *Pe*_p_ < 11 for the 0–35 mol % EGDMA samples, suggesting that all film formation is occurring inside the regime in which particles are predicted to accumulate at the top surface during drying, since *Pe*_p_ > 1. In conclusion, skin formation is expected from the model when the particles can coalesce, which explains the slowing rates of water loss with a decreasing EGDMA concentration.

### Film Microstructures

**Figure 4:**
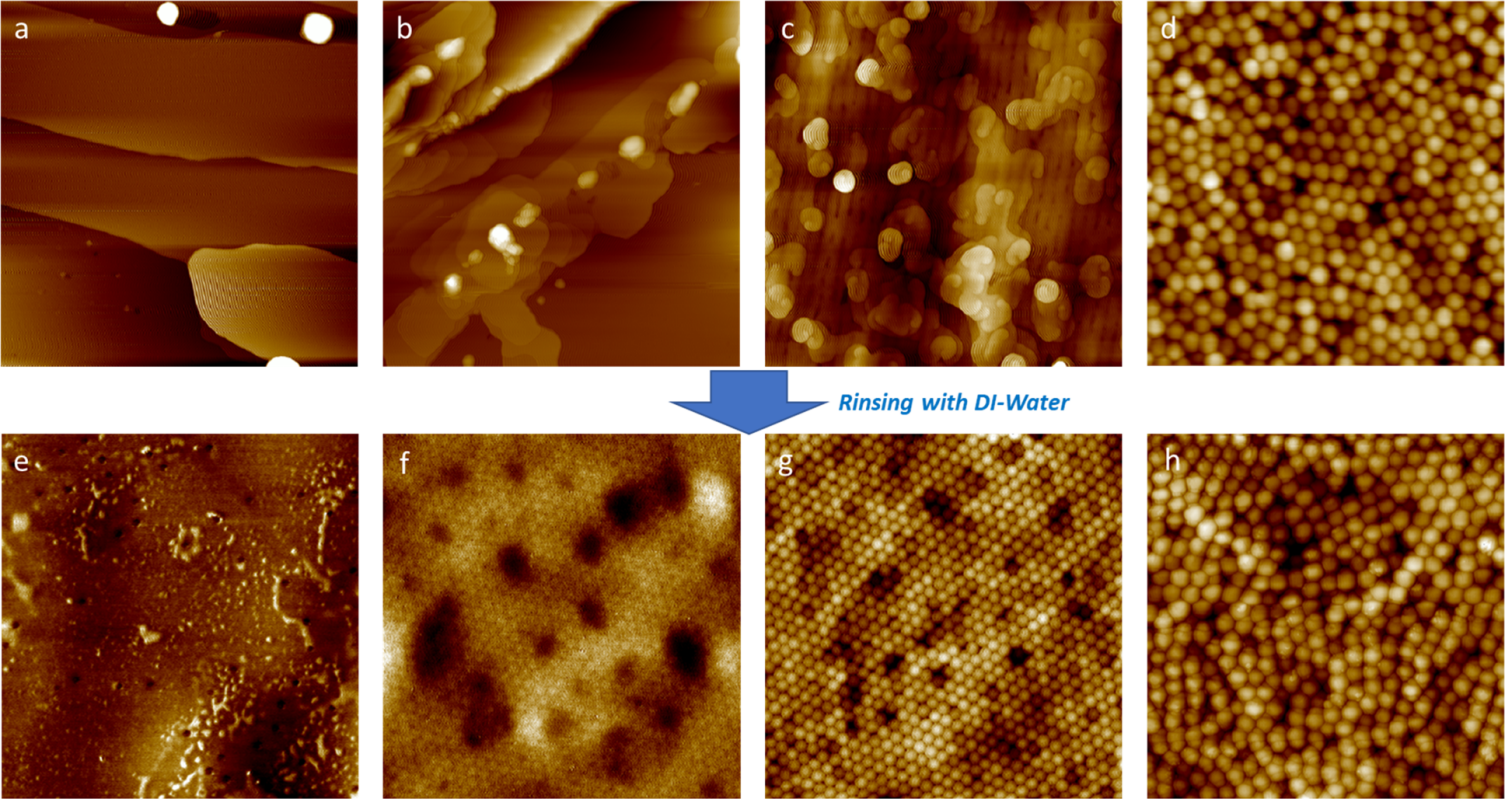
Representative AFM height images for as-cast (a–d) and rinsed (e–h) film surfaces with increasing cross-linker concentrations: (a, e) 0 mol %; (b, f) 10 mol %; (c, g) 15 mol %; and (d, h) 20 mol % EGDMA. These same samples were probed using RBS and ERD. All image sizes are 5 μm × 5 μm.

AFM is a useful way to determine the surface structure of latex films, as it allows for visualization of any surface features, such as uncoalesced polymer particles or surfactant, as have been studied previously.^10,13,17,64^ AFM images for a selection of four sample surfaces, before and after rinsing with water, are shown in Figure 4. Samples were rinsed for 30 s under a steady flow of deionized water from a water bottle while they were being tilted at an angle of 45°. Any water-soluble species present on the surface (such as surfactant or free initiator) is expected to be rinsed away.^10^ Rinsing was not possible for the 35 mol % EGDMA sample. Because there was no deformation or coalescence of the glassy particles, the film disintegrated upon contact with water. The as-deposited sample (prior to rinsing) showed broadly the same structure as for 20 mol % EGDMA in Figure 4d.

The AFM image for the 0 mol % EGDMA cross-linker sample (Figure 4a) shows smooth and flat regions at the surface with a stepped structure, possibly indicating a very thin layer of surfactant on the surface. When rinsed (Figure 4e), the initially smooth surface is replaced by a surface with inhomogeneities, such as small pits and bumps, that are ∼100 nm in size, comparable to the size of the colloidal particles. The bumps on the surface could be residues of what was not removed fully by rinsing or else colloids protruding from the surface. However, across most of the surface, individual particles cannot be identified. Surfactant could have existed in narrow channels further into the film but has been rinsed away to leave the pits that are observed in the images. Despite the interpretation of skin formation on the surface of the drying film (because of the low λ), some channels appear to have remained open. Surfactant on the particle surfaces might desorb during coalescence, some of which could be trapped in small pockets between particles near the surface before being rinsed to leave a pit. In summary, with the particles showing evidence for deformation and coalescence across broad regions of the surface, the structure is consistent with being in the wet sintering regime.

Next, the as-deposited 10 and 15 mol % EGDMA cross-linker films presented in Figure 4b,c are considered. The surface coverage in Figure 4c shows some island formation, which is indicative of water-soluble species being present on the surface, as has been seen in previous studies and is typically attributed to surfactant.^65−68^ These islands are between 20 and 140 nm thick, on top of the packed particle array. In AFM phase images (not shown here) the species in the island exhibit contrast to the particles, suggesting they differ in composition. When rinsed, the surface coverage on the 15 mol % EGDMA surface is removed and replaced by a distinctly defined array of deformed colloidal particles. The coverage on the 10 mol % sample is also removed, but the underlying particles are much less distinct in the image, with only faint particle boundaries being visible. These particles have deformed to produce a dodecahedral structure, as is typical for close-packed, deformed particles.^58^ More particle deformation takes place in the less cross-linked 10 mol % EGDMA film. This supports the idea (suggested by Brown’s elastic criterion^26^) that these particles experienced capillary pressure. The structure is consistent with the 10 and 15 mol % EGDMA colloids being in the capillary deformation regime. The fact that the initial surface coverage was removed by rinsing is consistent with it being surfactant (and possibly initiator fragments too).

Figure 4d shows the AFM height image of the top surface of a 20 mol % EGDMA sample. Distinct spherical particles are visible on the surface, with no apparent deformation having taken place following the close packing of the particles. Upon rinsing of the 20 mol % sample, as shown in Figure 4h, the same surface structure is visible. There is no evidence from microscopy for excess surfactant on the surface.

The absence of particle deformation with 20 mol % EGDMA cross-linker rules out the possibility of wet sintering or capillary deformation as the dominant deformation mechanism. As the particles are elastic, they are not expected to flow in a dry sintering mechanism but could potentially be elastically deformed. The shear modulus, *G*, for an elastomer with a density of strands between cross-links of ω is given by classical rubber elasticity theory as
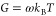
7

The network formed in the emulsion polymerization will exhibit randomness, which makes it impossible to know the value of ω with precision. By assuming each cross-link point connects two chains (and hence four strands), we estimate *G* on the order of 10 MPa, above the Brown criterion^26^ for capillary action of *G* < 9 MPa; therefore, particle deformation is predicted to be negligible. Because the particles in this case are cross-linked to a high degree, and *G* is high, eq 6 yields ϕ = 0.65 (random packing). In relation to the Routh–Russel deformation map, the particles are comparable to glassy particles with λ ≫ 10^4^. The nanostructures of the elastic particles (>15 mol % EGDMA) will be comparable to a very high value of λ (dry sintering or non-film-forming).

### Summary of Deformation Regimes

For films containing low levels of cross-linker, wet sintering is the likely mechanism, given the estimate for λ of 6 × 10^–4^ and observation of slowed water loss. For medium cross-linker (10 and 15 mol % EGDMA), some deformation is seen to occur, producing a honeycomb-like structure beneath a surface layer of surfactant. This suggests that capillary action (1 < λ < 100) is the driving force behind deformation, with narrow channels between deformed particles providing a pathway for surfactant transport to the top surface. Finally, for highly cross-linked films (>15 mol % EGDMA), as was expected from the space filling estimates, no deformation is occurring, leaving an open film structure which allows for the rapid evaporation of water from the film. No evidence for a surface excess of surfactant is found in AFM analysis. It is expected that these samples are at least within the receding water front regime^19^ and are non-film-forming as the cross-linker concentration is increased.

### Surfactant Distributions from Ion Beam Analysis

ERD and RBS^69,70^ have been conducted simultaneously on the samples, in order to determine the surfactant distribution in the top several hundred nm of the films both before and after rinsing with water. The same samples were analyzed by AFM, and the images are presented in Figure 4. In RBS, the energy of the backscattered ^4^He^+^ ion is used to determine the mass of the target atom, as well as its depth in the sample. ERD works similarly; however, the incident ^4^He^+^ ions forward-recoil hydrogen and deuterium atoms from the film. Energy is similarly used to determine the mass (and hence whether H or D) and the depth of the forward-recoiled atom. As deuterium is heavier than hydrogen, it is recoiled at a higher energy. In these experiments, only the surfactant (d-SDS) is labeled with deuterium, and therefore, ERD can determine with certainty the concentration profile of surfactant that is present near the surface of the film. When combined with RBS, which can detect heavier elements, such as Na and S in the surfactant, a full picture of the surfactant distribution near to the film’s top surface is gained.

There have been previous reports of the use of the ion beam technique of Rutherford backscattering spectrometry (RBS) for the analysis of surfactant concentration profiles. Lee et al. successfully employed RBS to analyze 30–50 nm thick surfactant layers on the surfaces of dry latex films and discovered that the type of surfactant influenced the thickness of any enriched layer,^15^ due to differences in the self-diffusivity of the surfactant, as was considered in their model. Slower diffusing surfactant led to greater accumulation at the top surface. Aramendia et al. also used RBS to study the differences between regular and reactive surfactant accumulation at the top surface, finding surfactant-enriched layers as thick as 100 nm on the top surface of films.^45^

ERD and RBS were performed on the full range of samples over two experimental runs. In the first run, 0, 10, 15, 20, and 35 mol % EGDMA samples were analyzed, both before and after rinsing with water. In the second run, samples containing 1, 2, 5, and 15 mol % EGDMA were analyzed using ERD and RBS. These samples were not rinsed and reanalyzed. The two sets of measurements were performed using the same incident beam and energy, incident and detector angles, and beam spot size. Because of some unavoidable differences in the experimental setup and data collection procedure (explained in the Supporting Information), the experimental spectra are not directly comparable. Nevertheless, the surfactant depth profiles can be compared.

**Figure 5:**
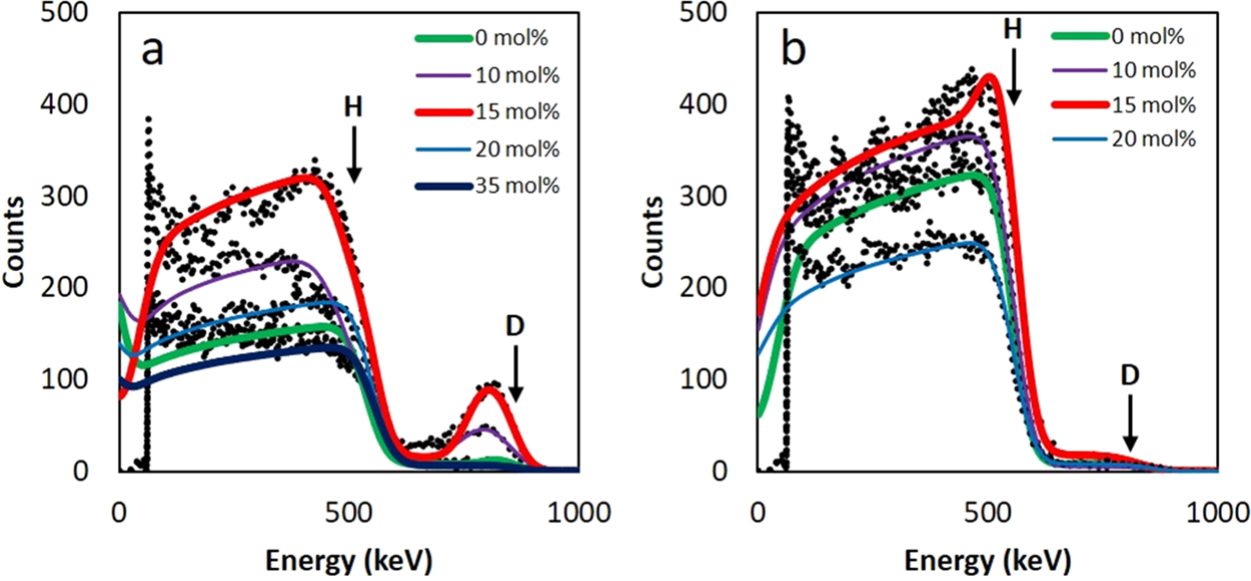
ERD experimental spectra and the best-fit models for the cross-linked films showing the hydrogen and deuterium counts near the top surface before (a) and after (b) rinsing with water. The data are shown with dashed black lines. The fits are overlain with colored lines, as are identified in the legend. The high counts at the lowest energies arise from forward scattered ^4^He^+^ ions that passed through the Al range foil and are not considered in the model.

The ERD spectra from the first run, before and after rinsing, are presented in Figure 5a and b, respectively. Corresponding RBS data for these experiments are presented in Figure S4. ERD data from the second run are shown in Figure S6. In ERD spectra, the numbers (counts) of forward recoiled H and D are plotted on the vertical axis against the energy of the ions incident on the detector on the horizontal axis. The technique measures the areal density (number per unit area) of elements in a layer. By assuming a mass density of 1 g cm^–3^ for the surfactant, initiator, and polymer, the energy is converted into a depth from the surface to yield depth profiles of the surfactant. Marked on Figure 5 are the scattering energies at which hydrogen and deuterium would be found if they were on the very top surface of the film. Counts below these energies correspond to atoms found beneath the film surface, since ^4^He^+^ ions lose energy as they travel deeper into the film, and the recoiled H and D lose energy as they travel through the film when leaving. Clear differences are seen in Figure 5 for films with increasing cross-linking concentrations and when comparing data from the rinsed and the as-deposited samples. Analysis of the rinsed 35 mol % EGDMA film was not possible as rinsing fully removed the film from the substrate.

For the study of surfactants, ERD is the more robust of the two IBA techniques, as it identifies deuterium, whose only possible source is the surfactant. RBS detects heavy elements, including sulfur, which has two possible sources: the surfactant and the initiator species. Furthermore, surfactant counterions (i.e., Na^+^) may not remain associated *with* the surfactant molecule. These considerations mean that relying solely on the RBS data is not sensible, and as such, the ERD has been used primarily but is complemented by the RBS (presented in Figure S4).

To model the film structure, the chemical formulas of each component (surfactant, initiator, and polymer) are used in combination with the known synthesis recipe to produce the approximate fraction of each element in the bulk composition: 33 atom % C, 10 atom % O, 53 atom % H, 4 atom % D, and trace amounts of Na, S, and K. Sublayers were added to the model of the film when a single bulk slab did not accurately model the experimental data. Different combinations of slab sublayers were trialed, including pure surfactant layers, polymer and ammonium persulfate initiator, and mixtures of these three, until the best fit was obtained by the minimization of the χ^2^ parameter. Enough sublayers were used to obtain a low χ^2^ value, and additional sublayers were not added if it did not significantly reduce the value further. The depth being probed by the beam is up to 1 μm in a dry film thickness of 75 μm. A full listing of the surface layer thickness, deuterium fraction, and surfactant concentration obtained from data fitting is shown in Table S1.

Using gravimetry and AFM, it has already been inferred that sparsely cross-linked particles are most likely in the wet sintering regime, medium cross-linked particles deforming in the capillary deformation regime, and highly cross-linked particles likely outside even the dry sintering regime. Indeed, the 35 mol % EGDMA particles are glassy at the film formation temperature.

In the film without cross-linking (0 mol % EGDMA), a very subtle peak of deuterium is visible. It was found to represent a 16 nm thick surface layer containing 95 mol % d-SDS. This result is corroborated by the RBS analysis, in which small peaks of Na, S, and K are seen at the top surface (Figure S4). The only possible source of the excess K is from the KPS initiator, suggesting that not just surfactant can be found at the surfaces of film, but also water-soluble initiator species. The layer contains 5 mol % KPS according to the data fitting.

In the medium cross-linked films (10–15 mol % EGDMA), obvious peaks of deuterium are visible in the spectra. This suggests a distinct layer of surfactant on the very top surface of the film, with a lower amount deeper down. For the 10 mol % EGDMA film, the best-fit model is a 127 nm thick surface layer containing 75 mol % d-SDS surfactant and 25 mol % KPS initiator. For the 15 mol % EGDMA film, the best-fit model was a 182 nm thick layer containing 95 mol % d-SDS and 5 mol % KPS. After the films were rinsed, as seen in Figure 5b, the deuterium peaks were removed for both the 10 and 15 mol % EGDMA samples. Then, the spectra resembled those without any surfactant excess prior to rinsing. This provides confirmation of the water-solubility of the surface layer, consistent with it being surfactant and/or initiator. These results agree with the RBS spectra.

The highly cross-linked films (20 and 35 mol % EGDMA) do not show any significant peak of deuterium in the ERD spectra. The best-fit models for both samples have a 4 or 5 nm thick surface layer of pure surfactant. For SDS molecules that are approximately 2 nm in length,^50^ this suggests either a surfactant bilayer on the surface, or simply a monolayer when surface roughness is considered. Surfactant monolayers are expected to be adsorbed on the surfaces of polymer particles^58^ following emulsion polymerization. There is no additional enrichment contributing to a surface excess layer. The low number of counts of deuterium represents the expected deuterium content in the bulk of the film. Likewise, the RBS spectra for both samples showed no excess of Na, S, or K.

In the second round of RBS and ERD, samples containing 1, 2, and 5 mol % EGDMA cross-linker were analyzed (Figure S6). In this lower range of cross-linker, thicker layers of surfactant enrichment were found on the surface but with a much-reduced surfactant concentration compared to the 10 and 15 mol % EGDMA cross-linker samples. That is, the surfactant was mixed within the polymer phase and not existing as a pure phase. An additional sample containing 15 mol % EGDMA was also analyzed in the second run, to compare with the 1, 2, and 5 mol % samples.

**Figure 6:**
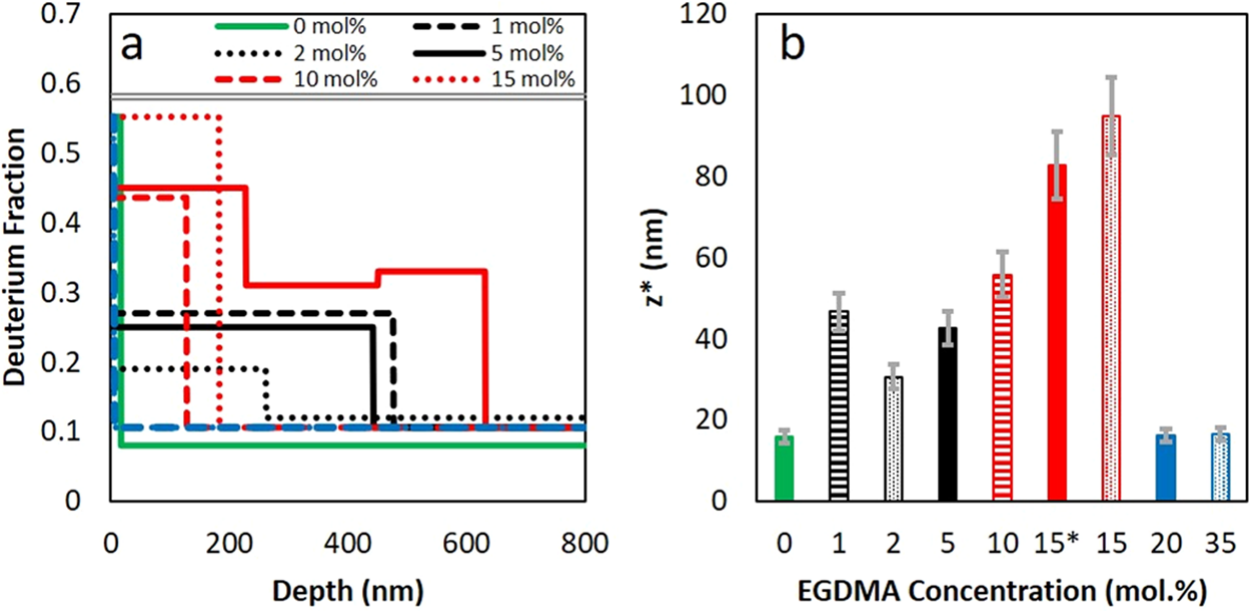
(a) Depth profiles of the deuterium fraction obtained from experimental data fitting. (b) Surface excess of surfactant, z*, calculated to depths of 200 nm. For both figures, with increasing concentrations of cross-linker, the colors are grouped together according to their EGDMA concentration as green (0 mol %); black (1–3 mol %); red (10 and 15 mol %); and blue (20 and 35 mol %). 15* designates data from the second run. Error bars represent a standard uncertainty of 10%, typical for layer thicknesses obtained using SIMNRA software.

To compare the distribution of surfactants across both runs, depth profiles of the deuterium fraction in the top 800 nm of each sample have been extracted from the best-fit models. A layer of pure deuterated surfactant has a deuterium atomic fraction of 0.58 (with C, O, Na, and S comprising the remaining fractions). To measure the extent of surfactant enrichment, the surface excess, z*, of deuterium has been calculated. This quantity is defined as the difference in the area under the deuterium fraction depth profile of a sample and that of a random mixture,^44^ here calculated to a depth of 200 nm:
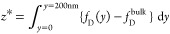
8where *f*_D_^bulk^ = 0.037 is the deuterium fraction in a random mixture, and *f*_D_(*y*) is the local deuterium fraction as a function of the depth, *y*. The depth profiles and corresponding deuterium excess for each sample are shown in Figure 6. There is an obvious trend in surfactant accumulation at the surface in relation to the cross-linker concentration. When the cross-linker concentration is either very low or very high, the surfactant is not enriched on the surface. In the intermediate range between these extremes, enrichment occurs, but the exact type of enrichment (pure surfactant layer or enriched polymer layer) depends on the cross-linker concentration as is explained hereafter.

**Figure 7:**
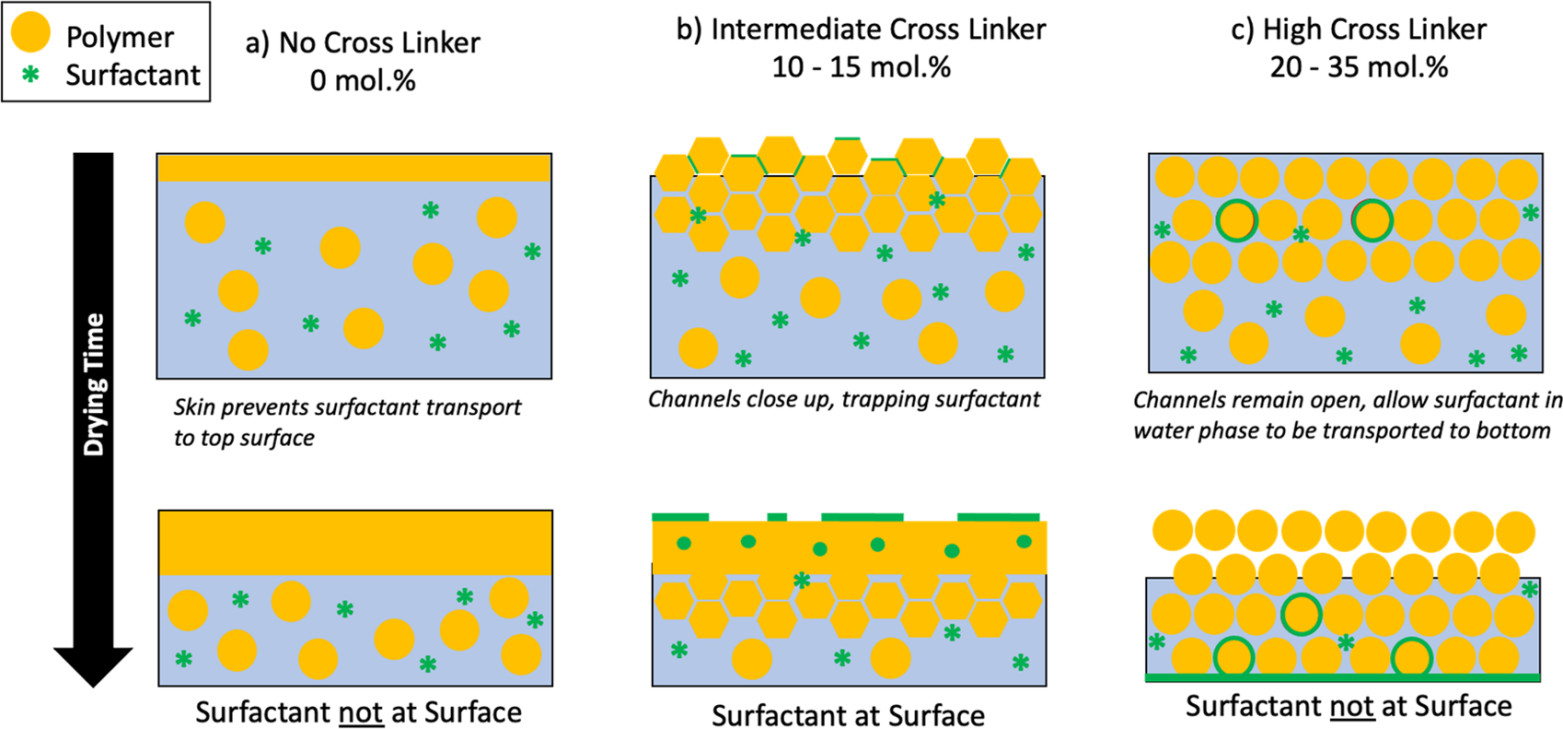
Schematic of the likely mechanisms of film formation for the cross-linked samples, and the corresponding surfactant distribution. (a) Films without cross-linker. (b) Films with 10 or 15 mol % EGDMA cross-linker. (c) Samples with 20 or 35 mol % EGDMA.

By approximating our cross-linker concentrations to Gromer’s^40^ deformation regimes, the results can be interpreted with reference to the model of particle deformation and the predictions of surfactant distribution. Figure 7 illustrates our interpretation of the data. With no cross-linking of the polymer chains, particles form skin layers, with an estimated value for λ of 1.8 × 10^–4^. These particles deform within the wet sintering regime. The skin layer acts as a barrier to surfactant transport to the top surface, leading to the low amounts of surfactant observed (Figure 7a). In experiments, the 0 mol % EGDMA film has a very low amount of excess surfactant with z* < 20 nm. Gromer et al.^40^ predicted a depletion of surfactant in this regime, which is not found here. The results can be understood by considering the likelihood of deforming particles forming a complete, cohesive skin layer (as was assumed in modeling work). There will undoubtedly be narrow channels through the skin that allow for some surfactant transport.

With medium levels of cross-linker (10–15 mol % EGDMA), particles experience deformation to produce a honeycomb-like structure under capillary pressure (equivalent to 1 < λ < 100). The narrow channels between the deformed particles act as a pathway for surfactant transport to the top surface of the film. As evaporation continues, the channels close, thus trapping surfactant between the particles, especially near the surface (Figure 7b). In ERD, there are distinct layers of almost pure surfactant (up to 95 mol %) with thicknesses up to 180 nm near the top surface, leading to 50 nm < z* < 100 nm.

With lower amounts of cross-linking (1–5 mol % EGDMA), some enrichment of surfactant near the surface of the film is seen. Thick layers (up to 500 nm) with surfactant concentrations of up to 40 mol % are found in ERD. It is suitable to describe these layers as being enriched in surfactant, rather than pure layers of surfactant. The surface excess is lower with a lower amount of cross-linking: 30 nm < z* < 50 nm.

For highly cross-linked films (≥20 mol % EGDMA), very little particle deformation is seen, as evidenced by the AFM. ERD finds a very low surface excess, with z* < 20 nm. The particles in these films have a higher elastic modulus than what would allow dry sintering (λ > 10^4^), but the surfactant results can be compared to the receding water-front regime analyzed by Gromer et al.^40^ The layer of particles remains nondeformed during drying, so that surfactant is not expected to be *trapped* between the particles. Instead, as the water level recedes below the film surface, surfactant in the water phase will be carried and deposited mainly near the bottom of the film (Figure 7c).

## Conclusions

This research has provided experimental evidence showing the profound influence of particle deformation on the distribution of surfactant in a colloidal film. The near-surface distribution of surfactant (and initiator species) was found using a combination of elastic recoil detection and atomic force microscopy on a series of colloids with increasing cross-link concentration.

The regime of particle deformation was deduced from consideration of the polymer mechanical properties and the water loss rates. In the wet sintering regime, which is represented by poly(butyl acrylate) particles containing no cross-linker, there was only a small amount of surfactant at the surface. As λ is increased by adding cross-links and hardening the polymer through copolymerization with EGDMA, and the capillary deformation regime is entered, a large amount of excess surfactant develops at the top surface during drying, as water is pinned at the film surface by capillary forces. Layers of nearly pure surfactant up to 180 nm thick were observed. Finally, when enough cross-linker was added to the system, pushing into the receding water-front regime and beyond (to non-film-forming in the case of 35 mol % EGDMA), no enrichment of surfactant at the surface was observed, except for a surfactant monolayer (or bilayer). These results provide the first systematic test of the cellular automata model proposed by Gromer et al.^40^ The experimental results showing the surfactant concentration at the surface follow the same trends as the model results in Figure 1.

The results show that the distribution of surfactant in colloidal polymer films is strongly dependent on the deformability of the polymer particles, as seen here through tuning of the particle deformation via the cross-linker concentration and hardening through copolymerization. The ability to control the distribution of surfactants in films for adhesive and coatings applications is critical to producing highly effective properties and preventing unwanted surfactant migration. Understanding from this research will inspire strategies to reduce surfactant enrichment at the surface of waterborne films.

## Supporting Information Available

Experimental details of ion beam analysis, mechanical analysis and resulting data, RBS data, ERD data from the second run, and best-fit parameters (PDF)
