# Supplementary material for: How Particle Deformability Influences the Surfactant Distribution in Colloidal Polymer Films
Source: Langmuir. 2022 Oct 4;38(41):12689–701. doi: 10.1021/acs.langmuir.2c02170 (PMC9583616; doi:10.1021/acs.langmuir.2c02170)
Supplement: Supplementary file 1 — la2c02170_si_001.pdf [file la2c02170_si_001.pdf]

Supporting Information

How Particle Deformability Influences the Surfactant Distribution in Colloidal Polymer Films

Toby R. Palmer[1], Hanne M. van der Kooij[2], Rohani B. Abu Bakar[1], Mathis Duewel[3], Katja Greiner[3], Callum D. McAleese[4], Pierre Couture[4], Matthew K. Sharpe[4], Richard W. Smith[4], Joseph L. Keddie[1]**

[1]Department of Physics, University of Surrey, Guildford, Surrey, GU2 7XH, UK

[2]Physical Chemistry and Soft Matter, Wageningen University, Wageningen, The Netherlands

[3]Synthomer Germany GmbH, Werrastraße 10, 45768, Marl, Germany

[4]Surrey Ion Beam Centre, University of Surrey, Guildford, Surrey, GU2 7XH, UK

**Corresponding Author: j.keddie@surrey.ac.uk*

S1. Ion Beam Analysis Energy Calibration

ERD:

The detector and beam set up is shown in **Figure S1**.

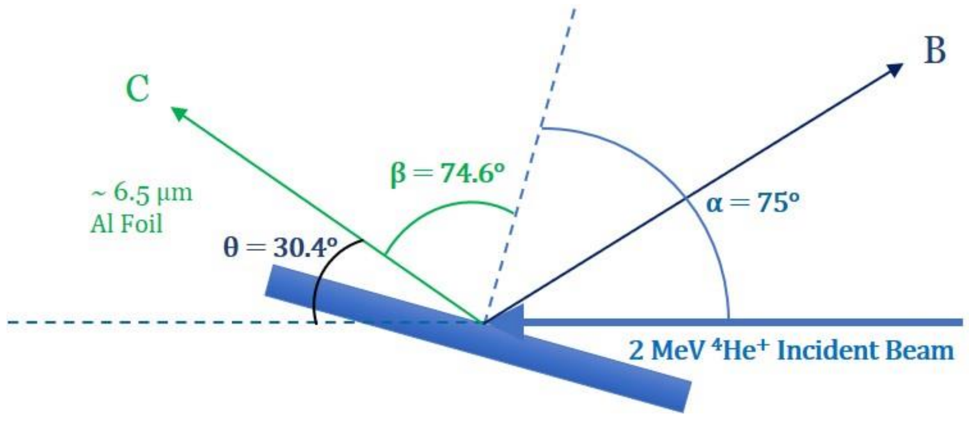

Figure S1. The detector and beam set up for the ERD and RBS measurements. The beam is incident on the sample at an angle of 75° to the normal. Detector B was used for RBS and detector C for ERD. There is a 6.5 μm range foil in front of detector C to stop or slow forward scattered He ions, thus preventing them from interfering with the H and D counts.

To determine the energy calibration for ERD measurements, spectra of two standard samples were taken, to determine the necessary parameters for analysis in SIMNRA. Thin films of hydrogenated poly(styrene) (hPS) and d8 poly(styrene) (dPS) (from Polymer Laboratories, Church Stretton, UK) were spin-cast from toluene onto silicon substrates.

To perform simulations, SIMNRA requires the product of the solid angle subtended by the detector, Ω and the number of particles incident on the sample, *N*, here given as *N*Ω. To find *N*, the obtained charge for a given sample is divided by the charge on an election, 1.6×10^{-19} C. For these standard samples, a value of *N*Ω that gave a good fit to the experimental data was used.

The standard samples were simulated by adjusting the energy per channel, calibration offset and detector resolution to obtain good agreement between the experimental data and the simulation for both hPS and dPS standards. The values were determined as:

Energy per Channel: 0.775 keV / Ch

Calibration Offset: 50 keV

Detector Resolution: 60 keV

Next, to determine the value of the solid angle for my experiments, a standard Kapton sample was used. Five 2μC spectra were collected and simulated using the energy calibration values found from hPS and dPS, and by adjusting *N*Ω until good agreement was seen. The solid angle, Ω for each run was calculated by dividing *N*Ω by the number of particles. It was observed that as the charge collected increased, Ω decreases, as shown in Figure S2.

Of course, Ω is a constant for the experiments, and so cannot truly be decreasing. In reality, what is changing is the number of forward recoiled particles, which are reducing as

more charge is collected. This is a result of beam-induced damage to the sample, reducing the number of hydrogen and deuterium atoms in the sample. To correct for this effect, a linear regression was fitted to the data in **Figure S2**, to establish Ω at an accumulated charge of 0 μC. This represents the undamaged sample. This approach yields a solid angle of 1.14 mSr.

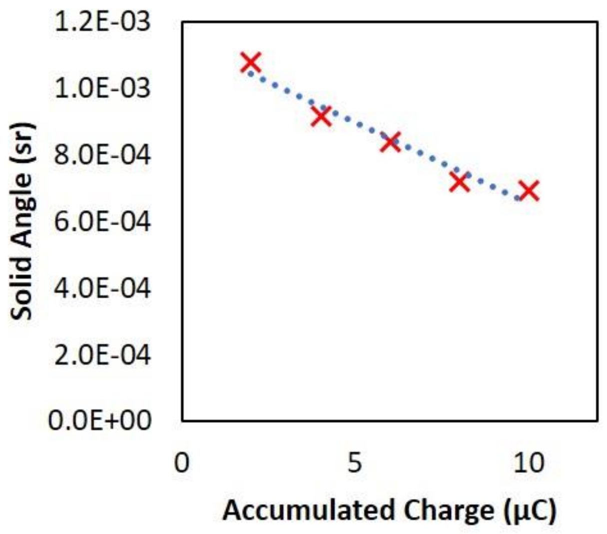

Figure S2. Calculated solid angle, Ω as a function of the accumulated charge, Q. The linear fit is given by the equation $\Omega = -0.00004819\ Q + 0.00114$.

RBS:

For the RBS energy calibration, a very similar process was adopted. Firstly, the energy per channel, calibration offset, and the detector resolution were found by fitting a standard sample containing Au, Ni, Cu, O and Si (the “AuNi” standard), while using an estimate for $N\Omega$. The solid angle was then determined using a second standard sample containing Si and trace amounts of Ar and As (the “SPIRIT” standard). In this case, the loss of heavier elements (C, O, Na, S, and K) was found to be insignificant, and so the linear regression required for ERD was not required. Energy calibration values were determined as (R1):

Energy per Channel: 1.74 keV / Ch

Calibration Offset: 30 keV

Detector Resolution: 25 keV

Solid Angle, Ω: 6.1 mSr

S2. Tensiometry and Mechanical Property Analysis

Surface Tensiometry. A drop shape analyzer (Krüss, FTA DSA1000B) was used for pendant drop tensiometry to measure the surface tension of cross-linked samples containing an additional 5wt.% surfactant. A drop with a volume of approximately 3 μL was injected and hung from a 0.87 mm diameter PTFE needle in the air. Then through analysis of the drop shape using the video camera system on the apparatus (FTA32 software) the surface tension was

determined. The experiments were conducted in a temperature-controlled room with a temperature of 22 °C. Sixty readings of each sample were taken, one second apart, with mean values reported.

Probe Tack Adhesion. Samples were cast using a cube applicator onto glass substrates, to achieve a wet film thickness of H = 100 µm. A fan-assisted convection oven (Heratherm, Thermo Scientific) was used for drying at T = 90 °C for a minimum of 3 minutes. Probe tack adhesion measurements were performed on a testing rig (Texture Analyzer, TA-XT Plus, Stable Micro Systems, Godalming, UK) using a spherical polypropylene probe (1 inch diameter), a load force of 4.9 N, a test speed of 5 mm/s, and a contact time of 1 s. Measurements were performed at 20.5 °C in a temperature-controlled room.

Stress-Strain Analysis. To prepare samples for mechanical analysis, thick films were cast into PTFE molds that were approximately 3.4 cm × 3.4 cm in size, and 6 mm deep. Initially, 9 mL was cast, and samples were left to dry at room temperature for one week. Then, a further 7 ml was deposited as a layer on top of the same sample, and again left to dry for one week. Following film formation, the films were removed from the molds and cut with a scalpel into strips measuring approximately 28 mm × 5 mm × 1 mm. The dimensions were measured using a digital caliper.

Testing was performed using a tensile apparatus (Texture Analyzer, TA-XT Plus, Stable Micro Systems, Godalming, UK), with each end of the sample held in clamps and with an initial gauge length between the clamps of 10 mm. The samples were strained at a constant speed of 0.5 mm/s until failure. The Young's modulus, Y was then obtained in the limit of low strain from the stress/strain curves, where the data fell approximately on a straight line. All experiments were carried out at a temperature of 20.5 °C in a temperature-controlled room.

Stress – strain curves obtained from probe tack and tensile experiments are shown for 0 – 15 mol.% samples in Figure S3. The Young's modulus, Y, was found from the linear gradient at low strains, as outlined in the main paper.

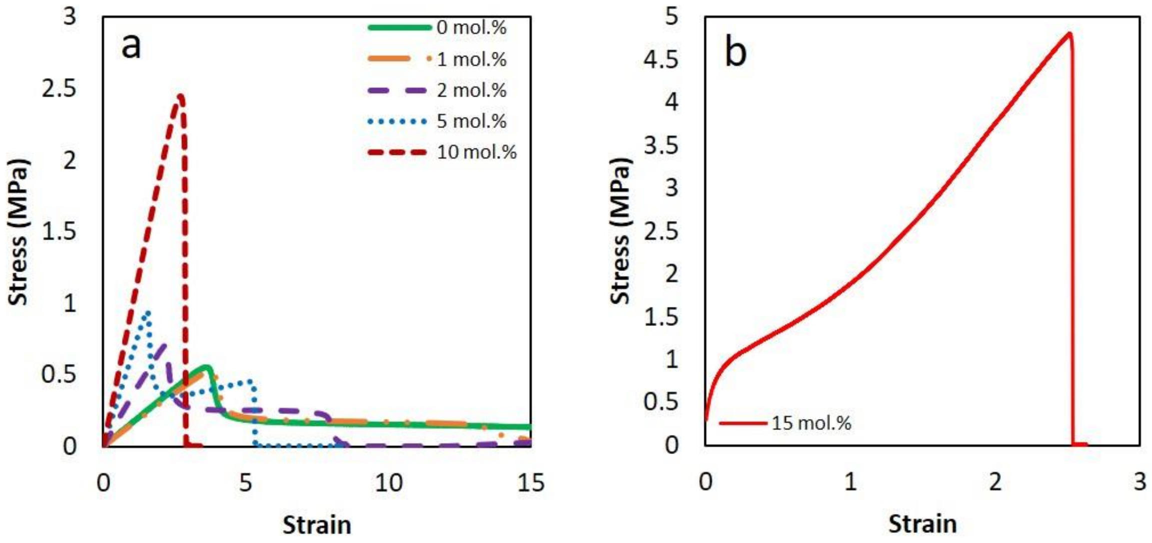

Figure S3. Stress/strain relationships for 0 – 15 mol.% samples obtained from (a) probe tack and (b) tensile experiments.

S3. RBS Data from the First Run

The equivalent RBS data has also been simulated, using the same layer compositions as for the ERD, but in some cases with small differences to the surface layer thickness required to fit the simulation to the data.

For as-deposited films, there are some clearly visible peaks of sodium, sulfur, and potassium, in contrast to no peaks once rinsed. This is consistent with the theory that water-soluble surfactant is being rinsed off the surface, as well as with the results of the ERD and AFM.

The presence of potassium in the RBS spectra gives more detail on the composition of the surface structure than is found in the ERD, as it suggests there is a small amount of initiator (KPS) mixed in with the surfactant layer. In the case of 0, 15, 20 and 35 mol.% samples, initiator makes up just 5 mol.% of the surface layer composition, with the remaining 95 mol.% coming from surfactant. For the 10 mol% sample, 25 mol.% of the surface is composed of the initiator, with 75 mol.% surfactant. The reason for this difference is not clear, although the shapes of the spectra for 10 mol.% in both the ERD and RBS spectra are consistent with other samples.

It is worth noting the presence of silicon in the spectrum of the rinsed 20 mol.% sample, which suggests that some of the sample substrate (silicon wafer) is being probed in the ion beam analysis. Upon inspection of the sample, it was observed that a small section of the film had been chipped away from the substrate, possibly due to damage from the He^+ beam. A small area of silicon wafer was exposed to the beam, leading to the observed silicon plateau in the RBS spectrum.

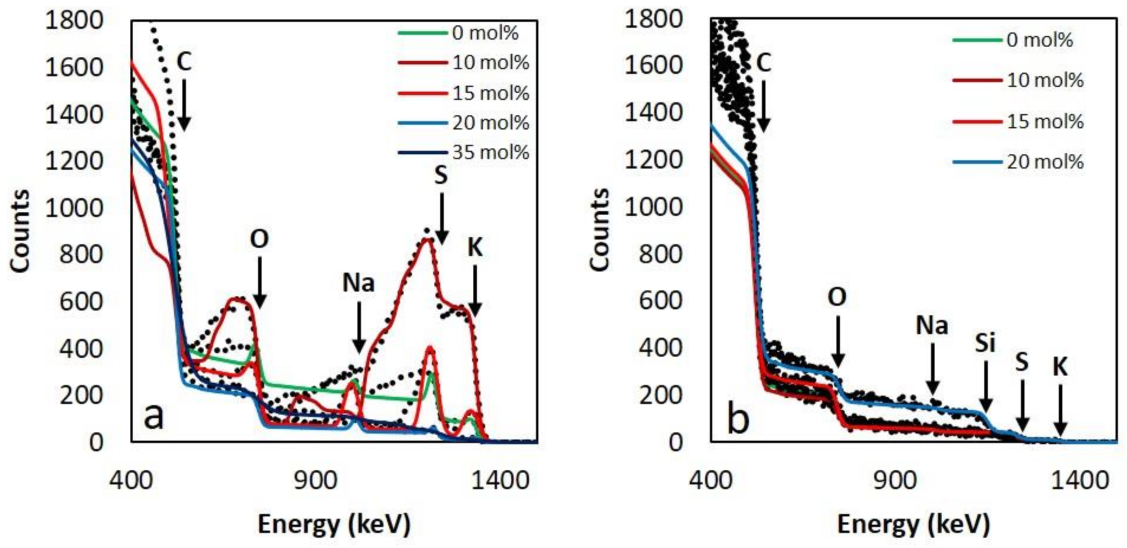

Figure S4. The RBS experimental spectra and the models for the best fits to the data for the cross-linked films before (a) and after (b) rinsing with water.

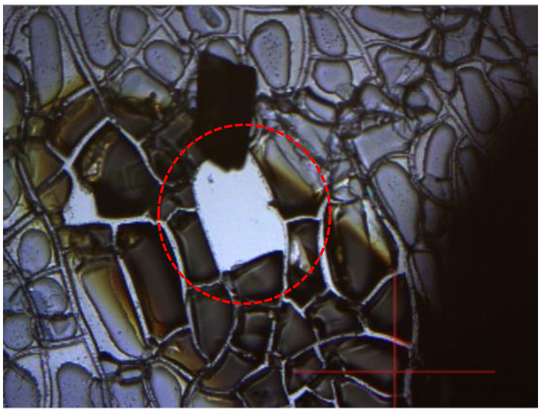

Figure S5. Optical microscope image of the 20 mol.% film showing the ion beam spot (dark region). The cracked surface structure is visible, with a section of film having been chipped off the substrate, exposing Si underneath. The chipped region is visible in Figure S4(b).

S4. ERD and RBS Data from the Second Run

The same energy calibration procedure was carried out for the second run of experiments as for the first, except for the solid angle determination for ERD measurements. In the second run of experiments, standard dPS and hPS thin film samples were used to determine Ω, instead of the Kapton standard. The values used are listed below.

ERD:

Energy per Channel: 0.29 keV / Ch

Calibration Offset: 60 keV

Detector Resolution: 80 keV

Solid Angle, Ω: 0.97 mSr

RBS:

Energy per Channel: 0.3 keV / Ch

Calibration Offset: 40 keV

Detector Resolution: 55 keV

Solid Angle, Ω: 6 mSr

Fitted spectra for the second round of ERD and RBS for the 1 – 5 mol.% cross-linker range are presented in Figure S6. The figure also includes data from the replicate sample (15 mol.%*). Results of the fitting are included in the main paper (Figure 6).

To allow a direct comparison of these samples to those analyzed previously, a repeat sample containing 15 mol.% cross-linker (identified as 15 mol.%*) was analyzed along-side these samples. This sample required a total of three surface layers (consisting of 75, 50 and 50 mol.% surfactant (with the remainder being polymer)), with a total thickness of 632 nm. This

surfactant profile is comparable to what was found in the 10 and 15 mol.% samples in the first run.

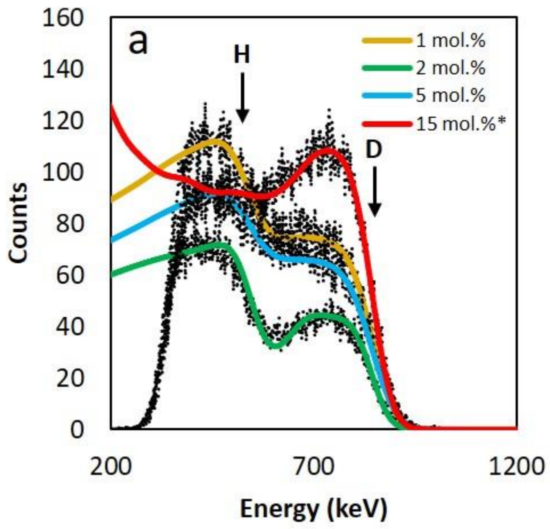

Figure S6. ERD and RBS experimental spectra and corresponding fits for the subsequent measurements on the 1, 2, 5 and 15 mol.%* samples. Experimental data are presented using the black dashed lines, with the simulations overlain as bold colored lines, as are identified in the legend.

S5. Ion Beam Analysis Differences

There are several unavoidable experimental differences between the two runs of Ion Beam Analysis experiments, which must be considered when comparing the data. In the first run, for ERD an analogue amplifier was used to process the detector signals but in the second run a digitizer was used. The digitizer is more affected by electronic noise in the system than the amplifier, thus leading to the cut-off at low energy of about 300 keV in the second run. A threshold was applied to the amplifier in the first run, with data acquired nearly down to 0 keV.

In the first run, a rectangular Hamamatsu PIN photodiode (3 mm × 30 mm area; model number S744/S3588-09) biased at 20 V was used as the detector in ERD. In the second run, a circular passivated implanted planar silicon (PIPS) detector (area of 150 mm^2, Canberra model number PD150-14-100AM) biased at 60 V was used. Both detectors were positioned approximately 30 cm from the sample surface. The choice of detector affects the energy resolution (and hence the depth resolution). Because of the two different shapes, the amount of beam straggling will also be impacted. The Al foil placed in front of the detector was changed for the second run, because of extensive damage over time, with a foil having a thickness of 8 μm. The foil thickness can influence the energy spread of the data. The data acquisition and goniometer motor software were updated between measurements. These factors could influence the height of peaks in the ERD spectra, as well as the noise associated with the data.

S6. ERD Analysis

Table S1. Best-Fit Parameters for the Surfactant Surface Layer Obtained from ERD Analysis.

Cross-Linker (mol.%)	Thickness (nm)	D Fraction	d-SDS Concentration (mol%)
0	16	0.55	95
1	476	0.26	40
2	261	0.17	25
5	442	0.24	40
10	127	0.44	75
15*	227	0.45	95
15	182	0.55	95
20	5	0.55	95
35	4	0.55	95
